# Liver fibrosis-4 score predicts outcome of patients with ischemic stroke undergoing intravenous thrombolysis

**DOI:** 10.3389/fneur.2023.1103063

**Published:** 2023-02-24

**Authors:** Davide Norata, Simona Lattanzi, Serena Broggi, Chiara Rocchi, Marco Bartolini, Mauro Silvestrini

**Affiliations:** Neurological Clinic and Stroke Unit, Department of Experimental and Clinical Medicine, Marche Polytechnic University, Ancona, Italy

**Keywords:** liver fibrosis, acute ischemic stroke, symptomatic intracranial hemorrhage, stroke prognosis, personalized medicine, thrombolysis

## Abstract

Some evidence suggests a possible influence of liver disease on stroke prognosis. We investigated the association between fibrosis-4 (FIB-4) score, a marker of liver disease, and the 3-month outcome in patients with ischemic stroke undergoing intravenous thrombolysis. We also evaluated the rate of symptomatic intracranial hemorrhage after thrombolysis. In this prospective cohort study, we enrolled consecutive patients with ischemic stroke treated with thrombolysis who had a 3-month follow-up. The FIB-4 score was calculated and the validated cut-off values were used to indicate high/low risk of advanced liver fibrosis. The primary outcome was 3-month poor prognosis estimated as a modified Rankin scale score ≥3. Of the 264 included patients, 131 (49.62%) had a 3-month mRS ≥3, with a significantly higher FIB-4 score, compared to those with a mRS <3 score (_adj_p <0.001). When adjusted for possible confounders by multivariate logistic regression, FIB-4 score remained a significant predictor of poor outcome (OR 1.894, *p* = 0.011), along with history of atrial fibrillation (OR 3.488, *p* = 0.017), admission NIHSS score (OR 1.305, *p* < 0.001), and low values of hemoglobin (OR 0.730, *p* < 0.001). Mechanical thrombectomy had a favorable effect on patients' outcome (OR 0.201, *p* = 0.005). The risk of poor 3-month outcome was significantly higher among the 32 patients (12.1%) with high risk of severe fibrosis (*p* = 0.007). FIB-4 score values were also related to symptomatic intracranial hemorrhage (*p* = 0.004), specifically among patients with high probability of advanced hepatic fibrosis (*p* = 0.037). FIB-4 score can be considered as a promising independent predictor of poor prognosis in patients with acute ischemic stroke undergoing intravenous thrombolysis.

## 1. Introduction

### 1.1. Background

According to the World Health Organization, 15 million people worldwide suffer from stroke each year. With a mortality rate of approximately one-third, it is the second most common cause of death and a leading cause of disability (1). Ischemic stroke is the most common type of stroke, accounting for approximately 80% of all acute strokes (2). Treatment approaches have been primarily directed at preserving neurons in the ischemic territory. The internationally approved treatments, recombinant tissue plasminogen activator (rt-Pa) and endovascular intervention, aim at rapid arterial recanalization to restore oxygen and nutrient supply to the affected area (3). Early recanalization after stroke is associated with a greater likelihood of favorable outcome (4, 5).

Liver fibrosis, the histologic precursor of cirrhosis, is a chronic disease (6), often preceded and promoted by an inflammatory process in combination with the accumulation of extracellular matrix in the liver (7). Several biomarkers have been proposed for the assessment of liver fibrosis. Among them, the fibrosis index (FIB)-4 has shown the best diagnostic accuracy for advanced hepatic fibrosis, as demonstrated by ultrasonographic studies in nonalcoholic fatty liver disease (NAFLD) (8, 9), the most common cause of liver dysfunction in Western countries (10). In recent studies, liver disease has been shown to be a strong predictor of both in-hospital and long-term mortality in stroke patients (11, 12). Moreover, it is independently associated with an increased risk of hemorrhagic complications (13), the most threated complication of intravenous thrombolysis, leading to poor outcome and increased risk of mortality (14). It is not yet clear whether these findings can also be applied to subclinical liver disease, which may not be uncommon in patients with stroke (15). In a recently published study, Fandler-Höfler et al. (16) showed that stroke patients with higher FIB-4 score values had worse clinical outcomes 3 months after mechanical thrombectomy but they didn't find any increased risk of postoperative parenchymal hematoma, hemorrhagic infarction and symptomatic intracerebral hemorrhage.

### 1.2. Objectives

The aim of the present study was to investigate the association of FIB-4 score with 3-month neurological outcome and symptomatic intracranial hemorrhage in patients with acute ischemic stroke treated with IV rt-Pa.

## 2. Material and methods

### 2.1. Study design, setting, and participants

We retrospectively identified consecutive patients admitted to the Stroke Unit of the University Hospital of Ancona, Italy, from January 2017 to April 2021 for acute ischemic stroke treated with IV thrombolysis. Each patient underwent routine blood sampling at admission (within 24 h of admission). Supplementary Table 1 provides an overview of the eligibility criteria.

The study was approved by the ethics committee of the Marche Polytechnic University (ID 57/2020) and conducted according to the Declaration of Helsinki. Informed consent was obtained from all subjects involved in the study or their representatives.

### 2.2. Variables

Demographics, medical history, National Institutes of Health Stroke Scale (NIHSS) scores (17), and admission blood pressure were documented at baseline. Laboratory tests [including serum levels of creatinine, glucose levels, hemoglobin (Hb), platelet count (PLT), absolute neutrophil count (ANC), absolute lymphocyte count (ALC), absolute monocyte count (AMC), alanine aminotransferase (ALT), aspartate aminotransferase (AST) total cholesterol, HDL cholesterol, LDL cholesterol, triglycerides, γ-glutamyltransferase (γGT), and creatine phosphokinase (CPK)] were determined by admission blood tests.

To quantify the extent of liver fibrosis, we used the noninvasive liver fibrosis score (FIB-4) for each patient at the time of admission.

The FIB-4 score was computed for every patient as follows:


$$\begin{array}{l} FIB4=\frac{Age\left(years\right)\times \;AST\left(\frac{IU}{L}\right)}{PLT\left(\times \frac{10^{9}}{L}\right)\times \sqrt{ALT\left(\frac{IU}{L}\right)}} \end{array}$$

As validated in previous clinical trials, prediction of advanced liver fibrosis was indicated using a cut-off value ≥2.67, whereas a score value <1.30 was used to exclude severe liver fibrosis with high probability (18, 19).

### 2.3. Outcome measures

The primary outcome measure was functional status at 3 months, evaluated in the hospital's outpatient setting. Because of its ease of use and interpretability, the modified Rankin Scale (mRS) is a widely applied clinical measure of global disability. In particular, it is used to assess recovery from stroke and as a primary end point in randomized clinical trials of stroke treatments. In our study, poor outcome was defined as the occurrence of death or major disability (mRS≥3) (20).

We also considered symptomatic intracranial hemorrhage (sICH) as a secondary outcome. We defined this hemorrhagic complication usually linked to rt-Pa, through the European Cooperative Acute Stroke Study (ECASS) III criteria, as follows (21). (1) Clinical deterioration: an increase of ≥4 points in NIHSS score or that led to death. (2) Radiographic features: any intracranial hemorrhage on CT/MRI performed at 22–36 h after stroke onset.

### 2.4. Biases and study size

**Figure 1:**
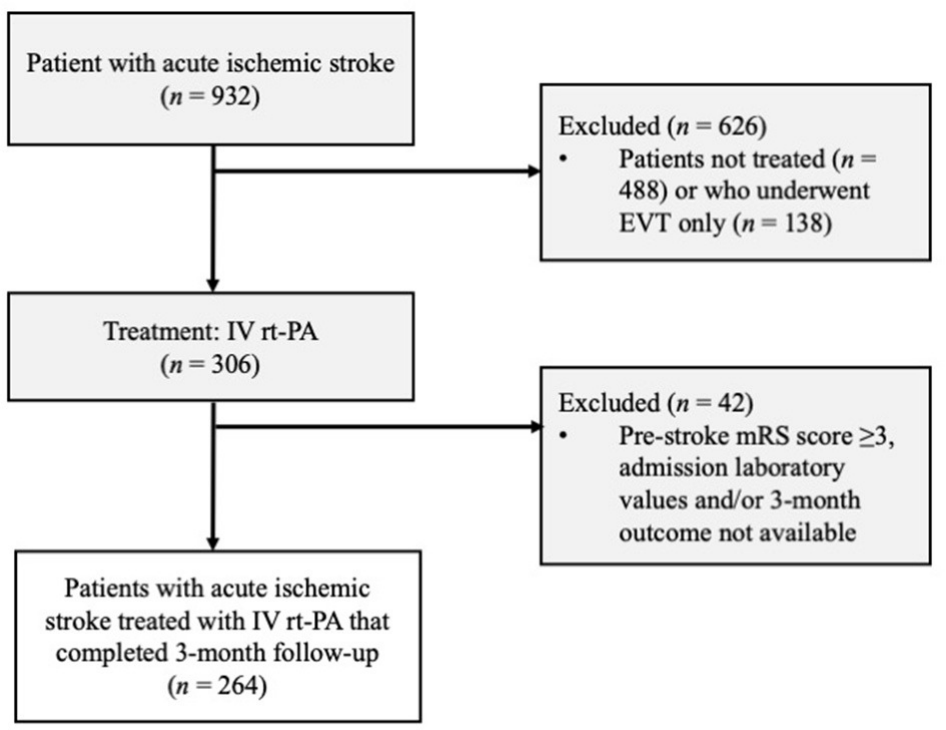
Patient selection flow diagram. EVI, endovascular treatment; IVT rt-PA, intravenous thrombolysis with recombinant tissue plasminogen activator; mRS; modified Rankin Scale.

We conducted this study on consecutive patients to avoid any selection bias. In order to address information bias, two aspects should be considered: the number of lost to follow-up was acceptable (Figure 1); the admission FIB-4 score was calculated only after the 3-month assessment, so the experimenter did not know the score value when assessing the 3-month mRS (primary outcome measure). Based on previous RCTs on Alteplase effectiveness (22), the minimum number of samples required to achieve a 95% confidence level with a marginal error of 0.05 was 241.

### 2.5. Statistical methods

We used standard statistical methods for descriptive statistics. Categorical variables were presented as frequencies and continuous variables as mean (standard deviation, SD) or median (interquartile range, IQR), when appropriate. Normality was assessed through the Shapiro–Wilk test. Depending on the normality of the distribution, comparisons were made by Student's *t*-test or Mann–Whitney test for continuous variables, and by Pearson χ^2^ test for categorical variables. The multivariate logistic regression was used to identify whether the FIB-4 score could be an independent predictor of poor 3-month outcome, and to establish the real prognostic value of demographic, clinical, and laboratory variables that reached statistical significance in the univariate analysis. To prevent biases, we did not include the variables already used for calculating the FIB-4 score in the logistic regression. An equivalent analysis was carried out for the secondary outcome, and is available in the Supplementary material. A two-tailed *p*-value of <0.05 was considered statistically significant for all tests. False discovery rate (FDR) correction was applied to deal with the multiple testing problem (results are expressed as adjusted *p* or _adj_*p*-values). Analysis was performed using JASP Team (2020). JASP (version 0.14.1).

## 3. Results

### 3.1. Participants and descriptive data

Of the 306 patients who suffered ischemic stroke treated with IV rt-Pa, 42 were excluded (Figure 1). Of the 264 enrolled patients, 131 (49.62%) had a modified Rankin Scale score of ≥3 after 3 months and 35 (13.3%) experienced a symptomatic intracranial hemorrhage (sICH; Table 1).

**Table 1:** Baseline characteristics according to the 3-month outcome.

**Baseline characteristics**	**Full cohort *n* = 264**	**mRS < 3 *n* = 133**	**mRS ≥3 *n* = 131**	***_*adj*_p-value***
**Demographics**				
Female sex	123 (46.6%)	53	70	0.074^a^
Age, years	69.3 (13.8)	65.9 (14.0)	72.7 (12.7)	< 0.001^b^^*^
**Clinical history**				
Hypertension	157 (63.3%)	73	84	0.080^a^
Diabetes mellitus	35 (14.6%)	18	17	0.963^a^
Actual smoking	59 (24.9%)	34	25	0.575^a^
Hypercholesterolemia	90 (36.6%)	51	39	0.337^a^
Atrial fibrillation	45 (19.2%)	11	34	< 0.001^a^^*^
Ischemic heart disease	31 (13.3%)	16	15	0.986^a^
Prior stroke	32 (13.5%)	18	14	0.116^a^
**Blood test variables**				
FIB-4 score	1.284 (0.989)	1.112 (0.734)	1.436 (1.186)	< 0.001^c^^*^
FIB-4 score ≥2.67	32 (12.1%)	9	23	0.021^a^^*^
FIB-4 score 1.31–2.66	95 (36.0%)	42	53	0.244^a^
FIB-4 score < 1.30	137 (51.9%)	82	55	0.004^a^^*^
Total cholesterol, mg/dl	176.1 (40.34)	178.0 (39.63)	174.2 (41.14)	0.653^b^
HDL cholesterol, mg/dl	51.76 (14.67)	53.21 (14.81)	50.24 (14.44)	0.233^b^
LDL cholesterol, mg/dl	103.47 (32.64)	105.24 (34.04)	101.59 (31.13)	0.575^b^
Triglycerides, mg/dl	96.00 (63.00)	96.00 (62.00)	95.00 (59.25)	0.760^c^
Creatinine, mg/dl	0.860 (0.330)	0.850 (0.265)	0.880 (0.425)	0.575^c^
Glucose, mg/dl	108.00 (45.00)	107.00 (43.25)	109.00 (44.00)	0.080^c^
Platelets, × 10^9^/L	207.00 (80.50)	207.00 (80.00)	207.00 (79.00)	0.768^c^
Hemoglobin, g/dl	13.05 (2.425)	13.40 (2.10)	12.60 (2.65)	< 0.001^c^^*^
ANC, × 10^9^/L	7.304 (3.338)	6.616 (3.228)	8.047 (3.312)	0.007^b^^*^
ALC, × 10^9^/L	1.670 (1.828)	1.722 (0.737)	1.614 (2.527)	0.768^b^
AMC, × 10^9^/L	0.697 (0.407)	0.642 (0.255)	0.757 (0.519)	0.090^b^
AST, U/L	17.00 (11.00)	16.00 (8.00)	19.00 (13.50)	0.021^c^^*^
ALT, U/L	22.00 (12.00)	22.00 (10.00)	22.00 (12.00)	0.768^c^
γGT, U/L	26.00 (24.00)	26.00 (23.00)	27.00 (23.00)	0.714^c^
CPK, U/L	99.00 (86.50)	94.00 (84.00)	108.50 (90.00)	0.389^c^
**In-hospital variables**				
Systolic blood pressure	139.0 (20.93)	139.0 (21.20)	139.0 (20.74)	0.986^b^
Diastolic blood pressure	76.4 (10.98)	76.4 (11.05)	76.4 (10.96)	0.986^b^
Admission NIHSS score	12.49 (6.16)	9.61 (6.17)	15.54 (4.49)	< 0.001^b^^*^
EVT	146 (55.3%)	60	86	< 0.001^a^^*^
sICH	35 (13.3%)	28	35	< 0.001^a^^*^

mRS, modified Ranking Scale; FIB-4, fibrosis score 4; ANC, absolute neutrophil count; ALC, absolute lymphocyte count; AMC, absolute monocyte count; AST, aspartate aminotransferase; ALT, alanine aminotransferase; γGT, γ-glutamyltransferase; CPK, creatine phosphokinase; EVT, endovascular treatment; sICH, symptomatic intracranial hemorrhage. Categorical variables were presented as frequencies and continuous variables as mean (SD) or median (IQR).

a χ^2^ test.

b Student t-test.

c Mann–Whitney test.

* adj p-value, p-values adjusted according to the false discovery rate (FDR) correction, < 0.05.

The mean FIB-4 score was significantly higher among patients with a poor prognosis compared with the other group (1,436 vs. 1,112, Student *t*-test −3.303, _adj_*p* < 0.001).

### 3.2. Main results

Demographic, clinical, and laboratory characteristics of patients are presented in Table 1. In univariate analyses, patients with poor prognosis more frequently had the following characteristics (Table 1): older age, history atrial fibrillation, high admission-NIHSS scores, high blood levels of ANC and AST, and low blood levels of Hb. As shown in Table 1, in the baseline study, adjuvant treatment with mechanical thrombectomy was associated with poor outcome.

We performed a multivariate logistic regression to assess the true predictive value of variables that apparently had an influence on prognosis on univariate analysis. SICH rates (Tables 1, 2) were not included in the calculation because they were not obtainable at baseline assessment. Although age and AST values were statistically significant in univariate testing, they neither were included in the logistic regression since they were already factored into the FIB-4 score, in order to avoid a distortion of FIB-4 score effect on prognosis (i.e., a confusion bias).

**Table 2:** sICH rates.

	**Full cohort *n* = 264**	**No sICH *n* = 229**	**sICH *n* = 35**	***p-*** **value**
FIB-4 score	1.284 (0.989)	1.249 (0.796)	1.697 (1.348)	0.004^c^^*^
FIB-4 score ≥2.67	32 (12.12%)	24	8	0.037^a^^*^
FIB-4 score < 1.30	137 (51.89%)	125	12	0.025^a^^*^

FIB-4, fibrosis score 4; sICH, symptomatic intracerebral hemorrhage. Categorical variables were presented as frequencies and continuous variables as mean (SD) or median (IQR).

a χ^2^ test.

c Mann-Whitney test.

* p-value < 0.05.

On multivariate analysis (Table 3), FIB-4 score (OR 1.894, *p* = 0.011), history of atrial fibrillation (OR 3.488, *p* = 0.017), high admission NIHSS score (OR 1.305, *p* < 0.001) and low blood values of Hb (OR of high Hb levels OR 0.730, *p* < 0.001) remained significant predictors of poor prognosis. In spite of what was hypothesized with the univariate analysis, the regression demonstrated a protective effect of thrombectomy (OR 0.201, *p* = 0.005). Other variables (female sex, and ANC) were not significant prognostic predictors.

**Table 3:** Logistic regression: FIB-4 score influence on 3-month mRS (primary outcome).

**Coefficients**	**Odds ratio**	***p*** **-value**	**95% confidence interval**	
			**Lower bound**	**Upper bound**
FIB-4 score	1.894	0.011^*^	1.160	3.094
Female sex	0.666	0.285	0.316	1.404
Atrial fibrillation	3.488	0.017^*^	1.253	9.710
Admission NIHSS	1.305	< 0.001^*^	1.177	1.448
EVT	0.201	0.005^*^	0.066	0.609
Hb, g/dl	0.730	< 0.001^*^	0.661	0.807
ANC, × 10^9^/L	1.000	0.223	1.000	1.000

mRS, modified Ranking Scale; FIB-4, fibrosis score 4 (continuous variable); EVT, endovascular treatment; ANC, absolute neutrophil count; Hb, hemoglobin.

* p-value < 0.05.

This statistical model showed good discriminatory power, with an area under the Receiver Operating Characteristic (ROC) curve of 0.877 (Supplementary Figure 1). It also produced a precision of 79.5% and an accuracy of 79.3%.

**Figure 2:**
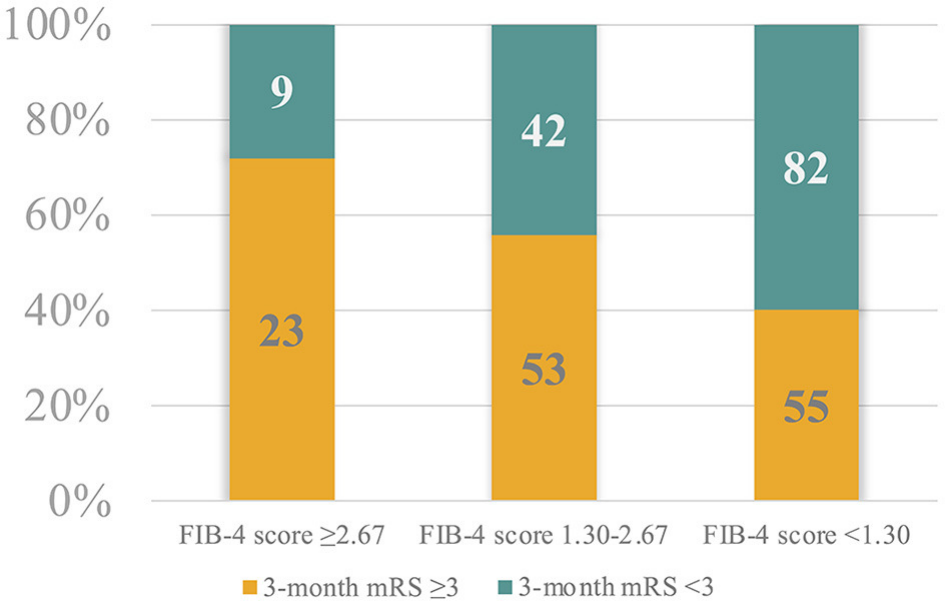
Patient proportional distribution based on the FIB-4 score cut-off values and the 3-month modified Ranking Scale.

Considering the FIB-4 cut-off values, we observed that the 32 patients (12.1%) with a high risk of advanced fibrosis (i.e., FIB-4 score ≥2.67) were more frequently associated with a poor 3-month outcome (_adj_*p* = 0.021), whereas the 137 patients (51.9%) with a high probability of exclusion of significant liver fibrosis (i.e., FIB-4 score <1.30) more frequently had a favorable 3-month outcome (_adj_*p* = 0.004; Figure 2).

As with the primary outcome, univariate analyses were used to investigate the influence of variables on sICH (Table 2 and Supplementary Table 2). Regarding the secondary outcome, our study showed a statistically significant relationship between rates of sICH and FIB-4 values (*p* = 0.004), admission NIHSS (_adj_*p* = 0.035), EVT (_adj_*p* < 0.001), and serum levels of glucose (_adj_*p* < 0.001) and ANC (_adj_*p* = 0.009). However, multivariate analysis only confirmed the effect of admission NIH score on the secondary outcome (OR 0.901, *p* = 0.035, Supplementary Table 3).

Moreover, we divided the entire study population according to the cut-off values of the FIB-4 score, and we noted that patients with FIB-4 score <1.30 (exclusion of liver fibrosis) had lower probability of sICH (FIB-4 score <1.30, *p* = 0.025), whereas ischemic lesions from patients with high risk of advanced fibrosis (i.e., FIB-4 values ≥2.67) tended statistically to bleed more frequently (*p* = 0.037).

## 4. Discussion

The extension of indications for intravenous rt-Pa in patients with stroke and, in particular, the lengthening of the time window, has stimulated the search for reliable predictors able to provide early information on the risk/benefit ratio of the treatment. The ability to predict the outcome shortly after hospitalization can play an important role in the decision-making process regarding the best therapeutic approach in stroke patients and to plan a proper overall therapeutic care. Recently, some of the main predictors of outcome in patients with ischemic stroke treated with rt-Pa have been described (23). High NIHSS scores, elevated systolic blood pressure values on admission, history of atrial fibrillation, and coronary artery disease were associated with poor outcome after 3 months. Another recent study on stroke patients undergoing rt-Pa reported that glycosylated hemoglobin blood levels were related to a poor early outcome but not to a poor functional prognosis at 3 months (24).

### 4.1. Interpretation of key results

In our study, in addition to clinical data, we considered using a simple and rapidly available index such as the FIB-4 score, based on laboratory parameters, to obtain prognostic information.

After adjustment for confounding factors by logistic regression analysis, we found that high values of FIB-4 score predicted outcome at 3 months in stroke patients treated with intravenous rt-Pa. Moreover, considering the validated cut-off values of this index, we were able to select a group of patients, characterized by a high risk of advanced liver fibrosis, who had a significantly higher probability of poor outcome than other patients. On the other hand, patients with exclusion of significant hepatic fibrosis had a higher probability of a favorable prognosis.

The FIB-4 score, which integrates blood levels of ALT, AST, and PLT, is not only a simple measure of patients' liver function but also reflects the complex systemic role of the liver itself. As highlighted by a cross-sectional study (11), liver dysfunction can lead to brain damage by several mechanisms, including small vessel disease or coagulopathy (25). In addition, NAFLD is associated with systemic inflammation (26, 27), vascular inflammation (28) and atherosclerosis (25, 29–33). Advanced liver disease is associated with mixed coagulopathy (34), which increases the risk of both thrombotic and hemorrhagic stroke.

It's intuitive that worse outcomes may be the consequence of higher comorbidity in general, not a worse effect of thrombolysis. The selection of outcomes more specifically linked to this treatment should be considered in further dedicated works. For that very reason, we introduced the evaluation of the symptomatic intracerebral hemorrhage, a crucial mechanism involved in modulating the prognosis of patients with ischemic stroke undergoing fibrinolysis. Although the multivariate analysis would seem to exclude a role for the FIB-4 score in predicting bleeding complications, this hypothesis could not be entirely ruled out for two reasons: the limited number of patients with symptomatic cerebral hemorrhage, and the statistical model's inability to corroborate data from previous studies, which have also constantly indicated that admission hyperglycemia plays a significant role in predicting post-thrombolysis intracranial hemorrhagic events (35–37). Our findings from univariate analysis suggested that being affected by severe hepatic fibrosis may increase the risk of intracerebral hemorrhage. Based on these findings, the poor outcome at 3 months in patients with advanced hepatic fibrosis may be, at least in part, related to hemorrhagic complications. As demonstrated in previous studies, the intravenous use of rt-Pa significantly increases the risk of intracranial hemorrhage, which is otherwise uncommon in ischemic stroke (38). Therefore, we hypothesize that for patients with severe hepatic fibrosis and ischemic stroke, the option for intravenous thrombolysis should be carefully evaluated considering the possible related risks.

In the present study, other indicators able to predict outcome at 3 months were identified. The negative prognostic role of atrial fibrillation in our patients was not unexpected although its significance has not been fully elucidated (39). In the Virtual International Stroke Trials Archive, no significant association was found between atrial fibrillation and overall stroke outcome (40). However, some studies found that atrial fibrillation was associated with favorable outcomes after thrombolysis for severe stroke, probably because of the effect of the thrombolytic agent on embolic arterial occlusion (37). In agreement with our findings, most studies suggest that atrial fibrillation may increase the risk of symptomatic intracranial hemorrhage and early death, and decrease the likelihood of favorable outcome after thrombolysis (41, 42).

Our finding of negative predictive effects of high NIHSS scores (23, 43–45) and low serum levels of hemoglobin (46–49) on outcome confirm previous findings in patients undergoing thrombolysis for stroke.

The results of our multivariate analyses showing a favorable effect of endovascular therapy on stroke outcome are consistent with the results of a recent systematic review of 19 randomized clinical trials (RCTs) (50). In this review, endovascular thrombectomy in patients with acute ischemic stroke due to occlusion of large arteries in the anterior circulation increased the chance of survival with good functional outcome (3-month mRS <3) with no negative effect on the risk of intracerebral hemorrhage or death. The predictive influence of anamnestic and laboratory variables on patients undergoing mechanical thrombectomy was recently investigated in a 2021 publication (51).

Toh et al. published an article at the beginning of 2023 addressing the same topic as the current study, with impactful results that confirm the significant influence of the FIB-4 score on the outcome of stroke patients undergoing thrombolysis in a highly representative sample of Asian population (52).

### 4.2. Strengths and limitations

Our study has some limitations. Because of its observational nature, this retrospective investigation does not reach the quality of evidence needed to draw definitive conclusions. Therefore, future prospective studies with established time points for blood sampling need to be conducted to assess the true cause-and-effect relationship between liver injury and stroke. In the event of a demonstration of a causal relationship, it will be critical to understand whether any improvement in liver condition can lower the risk of poor prognosis in stroke. Furthermore, it is not sufficiently clear whether the calculation of FIB-4 on admission can be considered reliable in expressing chronic liver damage, or is too influenced by stroke-related changes in blood levels of AST, ALT, and PLT. Further investigation is needed to obtain a clear answer with simultaneous assessment of the FIB-4 score and other markers of chronic liver disease.

On the other hand, the large sample of patients included and the easy usability of the score in a clinical setting with ordinary and cost-effective laboratory tests are the most important strengths of the study. We also used the already validated cut-off values of the FIB-4 score, that are strong indicators for the presence/absence of advanced liver fibrosis, significantly simplifying the calculation of the risk of poor outcome.

## 5. Conclusion

The results of the present study suggested that the FIB-4 score, a rapidly available and cost-effective parameter, can be considered as an independent predictor of poor prognosis, with high predictive accuracy, in patients with acute ischemic stroke undergoing intravenous thrombolysis.

In the new perspective of patient-centered medicine, identification of simple factors that predict treatment response is crucial to guide physicians in providing therapeutic strategies tailored to each single patient.

## Data availability statement

The raw data supporting the conclusions of this article will be made available by the authors, without undue reservation.

## Ethics statement

The study was approved by the Ethics Committee of the Marche Polytechnic University (ID 57/2020) and conducted according to the Declaration of Helsinki. Informed consent was obtained from all subjects involved in the study or their representatives.

## Author contributions

Conceptualization, software, formal analysis, writing—original draft preparation, project administration, and had full access to all the data in the study and takes responsibility for its integrity and the data analysis: DN. Methodology: SL. Investigation: DN, SB, and CR. Resources: SL and MB. Data curation: MB, DN, SB, and CR. Writing—review and editing: DN, SB, CR, and MS. Supervision: SL and MS. All authors have read and agreed to the published version of the manuscript.
