# Supplementary material for: Liver fibrosis-4 score predicts outcome of patients with ischemic stroke undergoing intravenous thrombolysis
Source: Front Neurol. 2023 Feb 24;14:1103063. doi: 10.3389/fneur.2023.1103063 (PMC9999710; doi:10.3389/fneur.2023.1103063)
Supplement: Supplementary file 1 [file Data_Sheet_1.docx]

Supplementary Material

# Supplementary Tables

| INCLUSION CRITERIA | EXCLUSION CRITERIA |
| --- | --- |
| Age ≥18 years of age | Pre-stroke disability corresponding to an mRS score ≥3 |
| Ischemic stroke diagnosis: acute onset of focal neurological deficit, lasting for ≥24h, OR acute brain infarction documented on brain CT/MRI | Other CNS disorders documented on brain CT/MRI |
| Stroke treatment with IV rt-PA according to the Italian 2016 SPREAD guidelines | History of stroke within 90 days before the clinical onset |
| Ability to collect admission laboratory values within 24 h from the clinical onset |  |

**Supplementary Table 1.** Inclusion and exclusion criteria. *Note:* mRS, modified Ranking Scale; CT, computed tomography; MRI, magnetic resonance imaging; IV rt-PA, intravenous recombinant tissue plasminogen activator; CNS, central nervous system

| BASELINE CHARACTERISTICS | **Full Cohort**  n = 264 | **No sICH**  n = 229 | **sICH**  n = 35 | ***_adj_P* value** |
| --- | --- | --- | --- | --- |
| **Demographics** | | | | |
| Female sex | 123 (46.6%) | 107 | 16 | 0.945 ^a^ |
| Age, years | 69.3 (13.8) | 68.9 (13.9) | 71.4 (12.6) | 0.795 ^b^ |
| **Clinical History** | | | | |
| Hypertension | 157 (63.3%) | 133 | 24 | 0.795 ^a^ |
| Diabetes Mellitus | 35 (14.6%) | 32 | 3 | 0.795 ^a^ |
| Actual Smoking | 59 (24.9%) | 49 | 10 | 0.795 ^a^ |
| Hypercholesterolemia | 90 (36.6%) | 76 | 14 | 0.795 ^a^ |
| Atrial fibrillation | 45 (19.2%) | 39 | 6 | 0.872 ^a^ |
| Ischemic Heart Disease | 31 (13.3%) | 25 | 6 | 0.795 ^a^ |
| Prior Stroke | 32 (13.5%) | 28 | 4 | 0.872 ^a^ |
| **Blood test variables** | | | | |
| Total cholesterol, mg/dL | 176.1 (40.34) | 176.7 (40.45) | 172.7 (40.06) | 0.822 ^b^ |
| HDL cholesterol, mg/dL | 51.76 (14.67) | 52.43 (14.47) | 47.44 (15.44) | 0.341 ^b^ |
| LDL cholesterol, mg/dL | 103.47 (32.64) | 102.95 (32.34) | 107.03 (34.99) | 0.822 ^b^ |
| Triglycerides, mg/dL | 96.00 (63.00) | 95.00 (62.75) | 98.00 (49.50) | 0.967 ^c^ |
| Creatinine, mg/dL | 0.860 (0.330) | 0.860 (0.340) | 0.880 (0.335) | 0.822 ^c^ |
| Glucose, mg/dL | 108.00 (45.00) | 106.00 (38.00) | 140.00 (54.50) | <.001*^c^ |
| Platelets, x10^9^/L | 207.00 (80.50) | 207.00 (81.00) | 200.00 (75.00) | 0.822 ^c^ |
| Hemoglobin, g/dL | 13.05 (2.425) | 13.10 (2.50) | 12.80 (2.20) | 0.795 ^c^ |
| ANC, x10^9^/L | 7.304 (3.338) | 7.049 (3.176) | 9.378 (3.945) | 0.009*^b^ |
| ALC, x10^9^/L | 1.670 (1.828) | 1.732 (1.918) | 1.168 (0.583) | 0.652 ^b^ |
| AMC, x10^9^/L | 0.697 (0.407) | 0.701 (0.415) | 0.668 (0.344) | 0.869 ^b^ |
| AST, U/L | 17.00 (11.00) | 17.00 (10.00) | 21.00 (20.00) | 0.129 ^c^ |
| ALT, U/L | 22.00 (12.00) | 22.00 (11.00) | 25.00 (11.50) | 0.665 ^c^ |
| γGT, U/L | 26.00 (24.00) | 26.00 (23.00) | 29.00 (34.25) | 0.795 ^c^ |
| CPK, U/L | 99.00 (86.50) | 97.00 (86.75) | 113.00 (90.00) | 0.822 ^c^ |
| **In-Hospital variables** | | | | |
| Systolic Blood Pressure | 139.0 (20.93) | 139.1 (20.61) | 138.0 (23.43) | 0.872 ^b^ |
| Diastolic Blood Pressure | 76.4 (10.98) | 76.5 (10.71) | 75.5 (12.91) | 0.822 ^b^ |
| Admission NIHSS score | 12.49 (6.16) | 12.06 (6.32) | 15.20 (4.23) | 0.035*^b^ |
| EVT | 146 (55.3%) | 117 | 29 | <.001*^a^ |
| **Supplementary Table 2.** Baseline characteristics according to the symptomatic intracranial hemorrhage (secondary outcome). *Note:* mRS, modified Ranking Scale; FIB-4, fibrosis score 4; ANC, absolute neutrophil count; ALC, absolute lymphocyte count; AMC, absolute monocyte count; AST, aspartate aminotransferase; ALT, alanine aminotransferase; γGT, γ-glutamyltransferase; CPK, creatine phosphokinase; EVT, endovascular treatment; sICH, symptomatic intracranial hemorrhage. Categorical variables were presented as frequencies and continuous variables as mean (SD) or median (IQR); ^a^, χ2 test; ^b^, Student t-test; ^c^, Mann-Whitney test; *, _adj_p value, p values adjusted according to the false discovery rate (FDR) correction. | | | | |

| **Coefficients** | **Odds Ratio** | ***P* value** | **95%Confidence interval**  **Lower bound Upper bound** | | | |
| --- | --- | --- | --- | --- | --- | --- |
| FIB-4 score | 1.062 | 0.799 | | 0.667 | 1.692 |  |
| Admission NIHSS | 0.901 | 0.035* | | 0.818 | 0.992 |  |
| EVT | 2.245 | 0.136 | | 0.774 | 6.506 |  |
| Glucose, mg/dL | 0.996 | 0.404 | | 0.988 | 1.005 |  |
| ANC, x10^9/L | 1.000 | 0.267 | | 1.000 | 1.000 |  |
| **Supplementary Table 3.** Logistic regression: factors predicting symptomatic intracranial hemorrhage (secondary outcome). *Note:* sICH, symptomatic intracranial hemorrhage; FIB-4, fibrosis score 4 (continuous variable); EVT, endovascular treatment; ANC, absolute neutrophil count; Hb, haemoglobin. | | | | | |  |

# Supplementary Figures


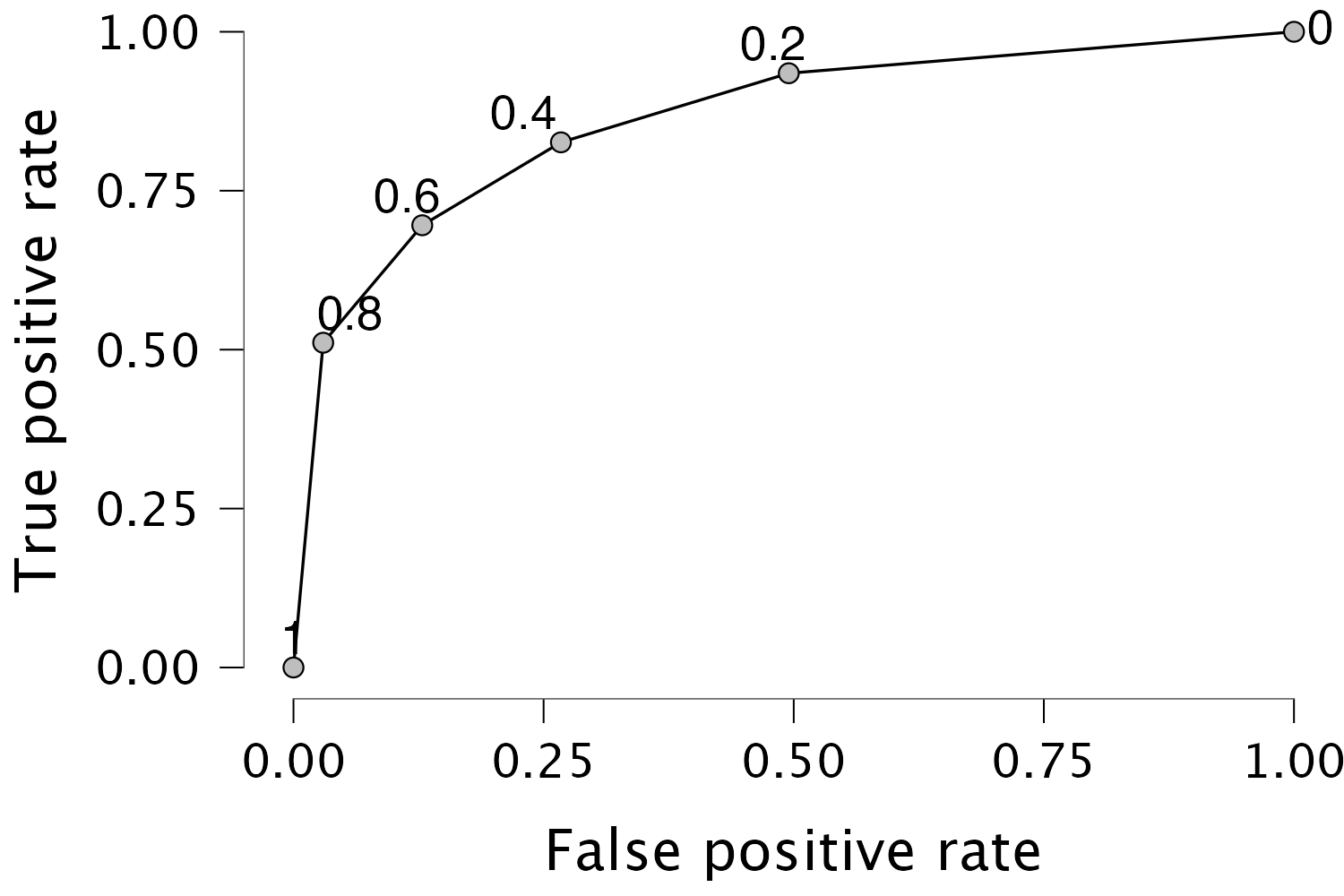


**Supplementary Figure 1.** Logistic Regression on factors predicting 3-month prognosis: Receiver Operating Characteristic (ROC) curve.
